# Modulatory Action by the Serotonergic System: Behavior and Neurophysiology in* Drosophila melanogaster*

**DOI:** 10.1155/2016/7291438

**Published:** 2016-02-17

**Authors:** Zana R. Majeed, Esraa Abdeljaber, Robin Soveland, Kristin Cornwell, Aubrey Bankemper, Felicitas Koch, Robin L. Cooper

**Affiliations:** ^1^Department of Biology, University of Kentucky, Lexington, KY 40506, USA; ^2^Department of Biology, College of Science, Salahaddin University-Erbil, Erbil, Iraq; ^3^Paul Laurence Dunbar High School, Lexington, KY 40513, USA; ^4^Veterinärmedizinische Fakultät, Universität Leipzig, 04103 Leipzig, Germany

## Abstract

Serotonin modulates various physiological processes and behaviors. This study investigates the role of 5-HT in locomotion and feeding behaviors as well as in modulation of sensory-motor circuits. The 5-HT biosynthesis was dysregulated by feeding* Drosophila* larvae 5-HT, a 5-HT precursor, or an inhibitor of tryptophan hydroxylase during early stages of development. The effects of feeding fluoxetine, a selective serotonin reuptake inhibitor, during early second instars were also examined. 5-HT receptor subtypes were manipulated using RNA interference mediated knockdown and 5-HT receptor insertional mutations. Moreover, synaptic transmission at 5-HT neurons was blocked or enhanced in both larvae and adult flies. The results demonstrate that disruption of components within the 5-HT system significantly impairs locomotion and feeding behaviors in larvae. Acute activation of 5-HT neurons disrupts normal locomotion activity in adult flies. To determine which 5-HT receptor subtype modulates the evoked sensory-motor activity, pharmacological agents were used. In addition, the activity of 5-HT neurons was enhanced by expressing and activating TrpA1 channels or channelrhodopsin-2 while recording the evoked excitatory postsynaptic potentials (EPSPs) in muscle fibers. 5-HT2 receptor activation mediates a modulatory role in a sensory-motor circuit, and the activation of 5-HT neurons can suppress the neural circuit activity, while fluoxetine can significantly decrease the sensory-motor activity.

## 1. Introduction

The fundamental mechanisms of modulating neural circuits in relation to specific physiological actions and behaviors in organisms are an area of current interest for basic research as well as clinical treatments [1]. Neural circuits change in activity by modulators such as serotonin (5-hydroxytryptamine, 5-HT) in altering sensitization in the gill-withdrawal reflex in* Aplysia* [2]. Biogenic amines can act as neurotransmitters and neuromodulators and are indispensable for the normal development of neural connections in organisms [3, 4]. Interestingly, the role of 5-HT as a modulator is evolutionarily conserved among organisms [5]. The serotonergic (5-HTergic) system plays many roles in* Drosophila* and other insects which are crucial for the animal survival and locomotion [6], feeding behaviors [7], learning and memory, aggression, circadian rhythms, sleep, heart rate, salivary secretion, insulin signaling, and synaptic transmission [8–17]. Being able to manipulate subsets of specific neurons in* Drosophila melanogaster* allows one to address the role of neurons associated with particular neuromodulators in altering specific behaviors [18–20]. In addition, the fruit fly nervous system is an attractive model to decode the neural circuits and interrogate the function of genes of interest in animal behavior [21, 22]. Also,* Drosophila* have a relatively simple 5-HTergic system that consists of 84 5-HTergic neurons in the larval central nervous system (CNS) [23, 24] and 106 5-HTergic neurons in adult CNS [23].

Serotonin is biosynthesized from an essential amino acid tryptophan, which is catalyzed by two enzymes, tryptophan hydroxylase (Trh) and aromatic-L-amino acid decarboxylase. After release of 5-HT into the synaptic cleft at nerve terminals by synaptic vesicles fusion, the action of 5-HT is terminated by its uptake into the nerve terminals by the serotonin transporter (SERT) [25]. SERT is an important target for many antidepressant agents [26].* Drosophila* genome contains a gene that encodes dSERT, which is homologous to the vertebrate human and rodent SERT (hSERT and rSERT). It has been shown that dSERT can be inhibited by a variety of chemicals, such as fluoxetine, which is a potent inhibitor of the SERT and is used as an antidepressant drug [27, 28]. The* Drosophila* genome harbors five different genes for the 5-HT receptors (5-HT1ADro, 5-HT1BDro, 5-HT2ADro, and 5-HT7Dro [11]) and the recently cloned 5-HT2BDro [9] receptor subtype. 5-HT receptors in* Drosophila* are G-protein coupled receptors (GPCR) [29], which are expressed in various neurons and selective regions of the nervous system which correlate with the 5-HT localization [14, 30].

Serotonin modulates locomotion activity in diverse groups of animals across the animal kingdom, for instance, in* C. elegans* [31, 32] and mammals [33]. Recently, it has been shown that various 5-HT receptors play a role in locomotive behavior in* Drosophila* [6]. Our results concur with this recently published study. In addition, we demonstrated that mutations in 5-HT receptor subtypes significantly reduce larval locomotion behavior. Moreover, we show that overactivation of 5-HT neurons in freely moving larvae and adults significantly compromises locomotor activity. At the cellular level, it was already shown that serotonin increases evoked motor unit activity which innervates body wall muscle fibers [34].

In this current study, we further investigated the 5-HT receptor subtypes that mediate the modulatory action of 5-HT in a sensory-CNS-motor circuit. 5-HT modulates the feeding behavior in mammals [35] as well as in insects. In honeybees, 5-HT increases the motility of the gut; however, it decreases feeding behavior drive in the CNS [7, 10, 14]. This indicates that 5-HT differentially changes feeding behavior. Also, it has been shown that if 5-HT2A receptor subtype is blocked, there is a decrease in feeding behavior in* D. melanogaster* [9]. 5-HT also decreases the feeding behavior in the ant* Camponotus mus* [36]. In this study, we address whether 5-HT alters mouth hook movements (MHMs), which is an assay for feeding behavior. This study demonstrates the role of 5-HT receptor subtypes and function of 5-HT releasing neurons in various behaviors within the ever increasingly invertebrate model* D. melanogaster*.

## 2. Materials and Methods

### 2.1. Fly Rearing and Stocks

Canton-S (CS) and w^1118^ flies were used as controls. Trh-Gal4 (II) (stock # 38388), Trh-Gal4 (III) (stock # 38389), D42-Gal4 (III) (stock # 8816), 5-HT1A-Gal4 (stock # 49583), 5-HT1B-Gal4 (stock # 46023), 5-HT2A-Gal4 (stock # 49574), 5-HT-7-Gal4 (stock # 49414), UAS-5-HT1B-RNAi (stock # 33418), UAS-5-HT2A-RNAi (stock # 31882), UAS-5-HT7-RNAi (stock # 27273), UAS-TrpA1 (stock # 26263), UAS-Trh (stock # 27638), Mi-5-HT1B (w^1118^; Mi{ET1}5-HT1B^MB05181^) (stock # 24240), PBac-5-HT2A (w^*∗*^; P{FRT(w^hs^)}2A P{neoFRT}82B PBac{GAL4D, EYFP}5-HT2A^PL00052^) (stock # 19367), Mi-5-HT2B (line 2) (w^1118^; Mi{ET1}5-HT2B^MB11858^) (stock # 29257), Mi-5-HT2B (line 1) (y^1^  w^*∗*^; Mi{MIC}5-HT2B^MI06500^) (stock # 40810), PBac-5-HT7 (w^1118^; PBac{WH}5-HT7^f05214^) (stock # 18848), and PBac-Trh (w^1118^; PBac{PB}Trh^c01440^) (stock # 10531) were obtained from the Bloomington* Drosophila* Stock Center (Bloomington, IN, USA). UAS-*shi* ^*ts1*^ was kindly provided by Kitamoto [37]. The flies were raised at room temperature (22-23°C), unless otherwise stated in figure legends, in vials containing cornmeal-agar-dextrose-yeast medium. Embryos or various staged larvae were collected for the experiments. The overexpressing and knockdown strains of the various proteins were not confirmed in this study with quantitative measures such as RT-PCR.

### 2.2. Chemicals

The agonists and antagonists used are defined for mammalian preparations and details of binding affinity and efficacy of the various* Drosophila* 5-HT receptor subtypes are not established. 8-Hydroxy-DPAT hydrobromide (5-HT1A agonist), CP 93129 dihydrochloride (5-HT1B agonist), *α*-methyl-5-hydroxytryptamine maleate (5-HT2 agonist), and AS19 (5-HT7 agonist) were purchased from Tocris Bioscience (Bristol, UK). 5-Hydroxytryptamine (5-HT), 5-hydroxytryptophan (5-HTP), fluoxetine hydrochloride,* para-*chlorophenylalanine (PCPA), 5-carboxamidotryptamine maleate salt, 5-methoxytryptamine, BW 723C86 (5-HT2B agonist), R(−)-DOI hydrochloride, (±)-DOI hydrochloride, 1-(3-chlorophenyl)piperazine hydrochloride (m-CPP), and LP-44 (5-HT7 agonist) were purchased from Sigma (St. Louis, MO, USA). All chemicals were dissolved in fly saline. However, AS19 and LP-44 were dissolved in dimethyl sulfoxide (DMSO). Fresh solutions of specific concentrations were made from stock solutions each time before starting an experiment. The DMSO concentration in AS19 was less than 0.01%. The DMSO concentration in LP-44 10 *μ*M, which is the highest concentration employed, was 0.1%.

### 2.3. Feeding of Compounds

Adult males and females were put inside a container with an apple-juice agar plate at the bottom. Specific drugs were dissolved in one milliliter (mL) of distilled water and mixed with 2 grams of cornmeal-agar-dextrose-yeast medium (shown in millimolar (mM) in the figure legends). Approximately fifteen (15) embryos were transferred from the apple-juice container to the drug mixture. The embryos were left to develop to the third-instar larval stage which were raised at room temperature (22-23°C), unless otherwise stated in figure legends. The same protocol was used for the control animals except that no drug was added to the food. It is known how much endogenous 5-HT concentrations are altered by feeding some of these compounds (50 mM PCPA, [38]); however, it is not known for other compounds we used such as the receptor agonists and antagonist. Such measures for each dosage and each compound are beyond the scope of these initial studies. Our approach was to provide varying dosages as a means to assess the potential varied concentrations on the effect of whole animal behaviors.

### 2.4. Behavioral Assays

#### 2.4.1. Locomotion Behavior in Larvae

Individual third-instar larvae were placed on apple-juice agar (1% agar) Petri dishes (about 8.5–9 cm diameter). The larvae were left inside the dish for one minute to acclimate to the environment, unless otherwise stated in figure legends. The body wall contractions (BWCs) were counted for one minute in a lightly illuminated environment at room temperature (22-23°C).

#### 2.4.2. Dominant Negative* shi* ^*ts1*^ and Larval Locomotion Behavior

The flies were raised at 23°C, 12 : 12 LD cycle. To obtain the locomotion behavior at room temperature (22-23°C) for control and* shibire* ^*ts1*^ expressing larvae, individual third-instar larvae were placed on the apple-juice agar. The larvae were left for 1 min to acclimate to the new environment, and body wall contractions were counted for the following 1 min. To obtain BWCs at restrictive temperature (37°C) for control and* shibire* ^*ts1*^ expressing larvae, larvae were placed inside a mixture of 0.5 gm fly food plus 0.5 mL water in a tube: 9.4 cm height, 2.4 cm top diameter, and 2.25 cm bottom diameter. The temperature range used was as previously used by Song et al. [39] for* shibire* ^*ts1*^. The larvae were incubated at 37°C for 20 min in the water bath. Individual larvae were placed on prewarmed apple-juice agar on a hot plate (30–32°C). The larvae were left for 1 min to acclimate, and the BWCs were counted for the following 1 min.

#### 2.4.3. TrpA1 Channels and Larval Locomotion Behavior

The flies were raised at 23°C, 12 : 12 LD cycle. To obtain BWCs at room temperature (22-23°C), individual larvae were placed on apple-juice agar at room temperature and left for 2 min to acclimate to the new environment. The BWCs were counted for 1 min in a lightly illuminated environment. To obtain locomotion behavior at restrictive temperature (32°C), individual larvae were placed on a prewarmed apple-juice agar plate on a hot plate (30–32°C). The larvae were left for 2 min. Afterwards, BWCs were counted for 1 min in a lightly illuminated environment. TrpA1 channels become activated at >28°C [40, 41].

#### 2.4.4. Larval Feeding Behavior

Individual larvae were placed inside a small Petri dish (5.5 cm diameter) that contained yeast solution (a few dried yeast granules were mixed with water). The larvae were left for one minute, and then the mouth hook movements (MHMs) were counted for one minute.

#### 2.4.5. Climbing Assay in Adults

The flies were raised at 23°C, 12 : 12 LD cycle. The adult flies were anesthetized by exposing them to CO_2_ and then males and females sorted out one day prior to performing the behavioral assay. Six to fourteen adult flies (2–6 days old), males and females, were tested separately. They were placed inside a tube: about 9.4 height, 2.4 cm top diameter, and 2.25 cm bottom diameter. During the experiment, the cotton plug was removed and another similar tube was placed over the top of the first tube. The bottom tube was marked at 8 cm height. The tube was tapped until all the flies fell into the bottom of the tube. A 10 sec period was set to observe how many flies would cross the 8 cm line in this period. This procedure was repeated 10 times, and there was one-minute interval between each trial in order for the flies to recover from first tapping. The climbing rate was calculated by combining all the fly cohorts that were tested at room temperature (modified from [42]). The same fly cohorts, which were used at room temperature climbing assay, were transferred into the water bath (37°C), and they were left for 10 min. After 10 min incubation, the flies were returned back to room temperature (22-23°C) to repeat the climbing assay. This procedure was repeated for 10 times and with a one-minute interval between each trial.

#### 2.4.6. Locomotion Activity in Adult Flies

Flies were raised at 23°C, 12 : 12 LD cycle. The adult flies, females or males, were anesthetized by exposing them to CO_2_. The flies were left to recover for at least 16 hours. Then, the flies (less than 1 to 6 days old) were anesthetized by cold. Adult flies were transferred into empty vials, and they were placed inside an ice bucket for 20–30 sec. The flies (4-5) were transferred into a plastic Petri dish (9 cm diameter and 0.8 cm height), and a glass cover was put over it. The flies were left for 10–13 min to recover from cold anesthesia at room temperature. Afterwards, the flies were placed in a 37°C room. The fly locomotion activities were recorded with a webcam (WEBCAM HD4110, Hewlett-Packard Company, Palo Alto, CA), which was connected to a computer, and the activity was recorded at 5 frames per second (5 fps) for 10 min using VirtualDub-1.10.4 software (http://www.virtualdub.org/). The locomotion activity was analyzed for two time points, for 10 sec at the 5th min and for 10 sec at 10th min using manual tracking (http://rsbweb.nih.gov/ij/plugins/track/track.html) in ImageJ software (1.47 v) (http://rsbweb.nih.gov/ij/download.html) [43].

### 2.5. Optogenetics Experiments

#### 2.5.1. Fly Lines

We used a recently created channelrhodopsin-2 (ChR2) line which is very sensitive to light: y^1^ w^1118^; PBac{UAS-ChR2.XXL}VK00018 (BDSC stock # 58374) [28]. Virgin females from UAS-ChR2-XXL were crossed with males of Trh-Gal4 (BDSC stock # 38389) line to express ChR2-XXL variant in serotonergic neurons. We also used UAS-H134R-ChR2; Trh-Gal4 (III) homozygous line, which is kindly provided by Dr. Schoofs et al. [44], for the electrophysiological study since the ChR2-XXL was too sensitive for the electrophysiology experiments.

#### 2.5.2. All-Trans Retinal (ATR) Preparation

All-trans retinal (500 mg; Sigma-Aldrich, St. Louis, MO, USA) was dissolved in 17.6 mL absolute ethanol to make a 100 mM stock solution. 100 *μ*L of 100 mM stock solution was transferred to small tubes and wrapped in aluminum foil, to prevent being exposed to light, and kept in −20°C freezer.

#### 2.5.3. Preparation of Fly Food Supplemented with ATR

In order to prepare fly food supplemented with 1 mM ATR, 10 mL fly food was dissolved in microwave. After the food was cooled, 100 *μ*L of 100 mM ATR was mixed well with fly food or 100 *μ*L of absolute ethanol (vehicle) was mixed with food as a control. The food vial was wrapped in aluminum foil until it was solidified. The flies were transferred to a vial containing ATR and kept in a dark place (to keep the ATR from degradation) at room temperature (22-23°C).

#### 2.5.4. Larval Locomotion Behavior

Locomotion behavior was assessed by placing a single larva on an apple-juice agar plate. The larva was left for one minute to acclimate to the new environment. The body wall contractions were being counted for one minute (BWCs/min) while the larva was being exposed to a dim white light. Also, body wall contractions were counted while the larva was exposed to focused focal blue light (a focused light through a microscope eyepiece with a mounted LED; see [45]).

#### 2.5.5. Negative Geotaxic Assay

The adult flies aged 2–8 days were anesthetized with CO_2_. The males and females are to be sorted out and transferred into separate vials in cohorts of 10–14 flies. The flies were left to recover for 24 hours before running the experiments. A plastic vial (*Drosophila* culture cylindrical vial 1-1/4′′ diameter × 4′′ tall) was marked at 8 cm length, and the 10–14 cohort flies were transferred to that empty marked vial. Another plastic vial was placed on top of the marked one (modified from [42]). The flies were left for one minute. The vials were tapped to knock down the flies to the bottom of the tube. Then, the number of flies which climbed across the 8 cm mark in 10 sec was recorded. This procedure was repeated before and after exposure to blue light.

#### 2.5.6. Recording Evoked Sensory-Motor Circuit Activity

Third-instar larvae were placed in the dissecting dish and pinned out on anterior and posterior regions with the dorsal side up. A modified HL3 saline (NaCl 70 mM, KCl 5 mM, MgCl_2_·6H_2_O 20 mM, NaHCO_3_ 10 mM, Trehalose 5 mM, sucrose 115 mM, Trizma acid 25 mM or BES 25 mM, and CaCl_2_·2H_2_O 1 mM, pH 7.1) was used as physiological saline [46]. The dissection procedures have been previously described [34]. The two last segmental nerves, which innervate last body segment, were cut close to the posterior end. Sharp microelectrodes (3 M KCl) were used for monitoring muscle fiber 6 or 7. The segmental nerves were stimulated at 40 Hz, 10 pulses (S88 Stimulator, Astro-Med, Inc., GRASS Co., USA). There was 10 sec delay from first stimulation to the next stimulation train. The excitatory postsynaptic potentials (EPSPs) were recorded with an AxoClamp 2B (Axon Instruments, USA), converted with a PowerLab, 4SP (ADInstruments, USA), and analyzed with LabChart 7.0 (ADInstruments, USA). The traces were measured by averaging the responses in 8 stimulations trains made with normal saline and 8 stimulations trains after exchanging saline with various compounds, unless otherwise stated in figure legends. The suction electrodes which were used to stimulate the segmental nerves in each preparation were slightly different; therefore, a range of stimulation voltages were used. Depending on how tight the seal is with the suction electrode and the nerve, the voltage must be adjusted to evoked action potentials in the sensory nerves. The current is not directly varied but is a reflection of the voltage and the seal resistance for each preparation.

### 2.6. Statistical Analysis

All data are represented as mean ± SEM. One-way ANOVA test was carried out for multiple comparisons among treatments. Bonferroni *t*-test was performed to obtain significant levels (*P* values) of various groups of flies (SigmaPlot version 12.0). Paired *t*-test was employed to compare the number of EPSPs between the saline and agonist or saline-containing compound. Student's *t*-test was used to compare the treatment and control groups. The level of significance (*P* value) less than or equal to 0.05 is considered statistically significant.

## 3. Results

### 3.1. Oral 5-HT Administration: Locomotion and Feeding Behaviors

**Figure 1:**
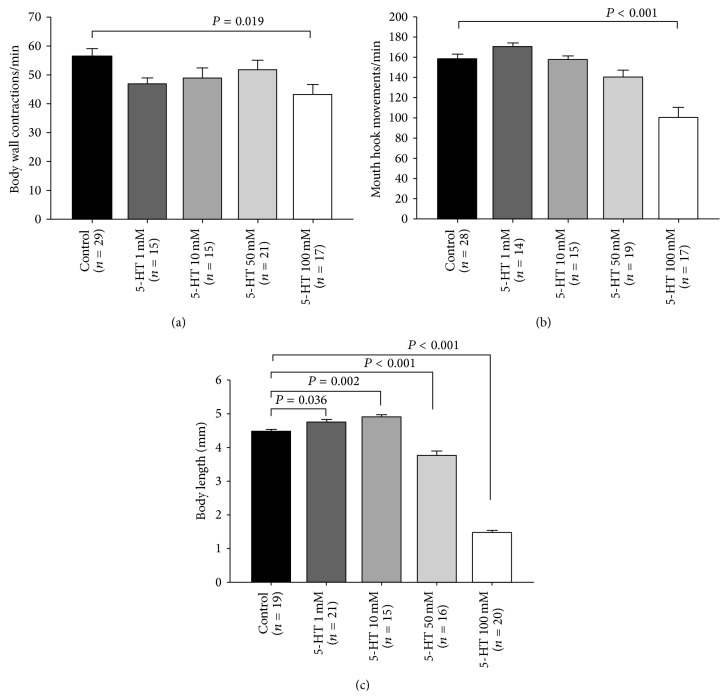
Serotonin modulatory action in feeding and locomotion behavior. (a) Effect of 5-HT on larval locomotion behavior. Canton-S (CS) larvae fed food containing 5-HT at 100 mM had significantly reduced body wall contractions. (b) 5-HT at 100 mM significantly reduced mouth hook movements. (c) 5-HT 1 mM and 10 mM significantly increased body length. However, 5-HT at 50 mM and 100 mM significantly reduced body length in comparison to control animals (CS). One-way ANOVA was used for multiple comparisons, and Bonferroni *t*-test was employed to compare treatments with control group. Data presented as mean ± SEM. *P* value < 0.05 is significant.

The rationale for feeding 5-HT as compared to injections is to reduce the handling stress associated with systemic injections. 5-HT was orally administered to the larvae at various concentrations: 1 mM, 10 mM, 50 mM, and 100 mM. The embryos were collected and were placed inside a mixture of 0.5 gm fly food and 0.5 mL water (control) or 0.5 gm food and 0.5 mL water plus various 5-HT concentrations. 5-HT significantly reduced the body wall contractions (BWCs) and mouth hook movements (MHMs) at 100 mM high dose (Figures 1(a) and 1(b)). 1 mM did not significantly alter mouth hook movements (MHMs); however, 100 mM significantly reduced the MHMs (Figure 1(b)). Also, 5-HT altered the developmental size of larvae. A low level of 5-HT (1 mM, 10 mM) resulted in significant increases in body length; however, at high concentrations (50 mM and 100 mM), the body length is significantly reduced compared to controls not fed 5-HT (Figure 1(c)).

### 3.2. Oral 5-HT Precursor (5-HTP) Administration: Locomotion and Feeding Behaviors

**Figure 2:**
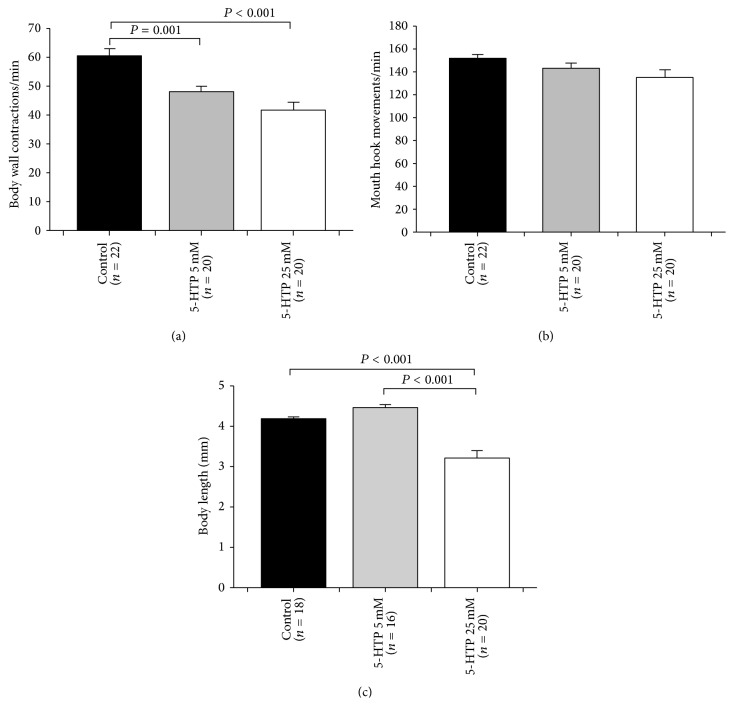
The effect of 5-hydroxytryptophan (5-HTP), a 5-HT precursor, on feeding and locomotion behaviors. (a) 5-HTP (5 mM and 25 mM) significantly reduced the body wall contractions in larvae. (b) When the larvae were fed on food mixed with 5-HTP, no significant difference was observed in feeding behavior in comparison to control animals (CS). (c) Body length significantly decreased at 25 mM 5-HTP. One-way ANOVA was used for comparison, and Bonferroni *t*-test was employed to compare treatments with control group. Data represented as mean ± SEM. *P* value < 0.05 is significant.

Another approach to perturb the 5-HT level in neurons and systemically is by providing more of the precursor to synthesize 5-HT. We predicted a similar result with feeding the precursor to feeding 5-HT itself to the larvae. The 5-HT precursor 5-HTP was orally administered during early stages of development. The embryos were collected and were placed inside a mixture of 0.5 gm standard fly food and 0.5 mL water (control) or 0.5 gm food and 0.5 mL water in addition to various concentrations of 5-HTP. 5-HTP at 5 mM and 25 mM reduced the BWCs in third-instar larvae (Figure 2(a)). On the other hand, there was no statistically significant reduction in MHMs (Figure 2(b)). Body length did not change for 5-HTP at 5 mM, but the lengths did decrease at 25 mM as compared to controls and ones fed 5 mM (Figure 2(c)).

### 3.3. Oral PCPA Administration: Inhibition of Tryptophan Hydroxylase (Trh)

**Figure 3:**
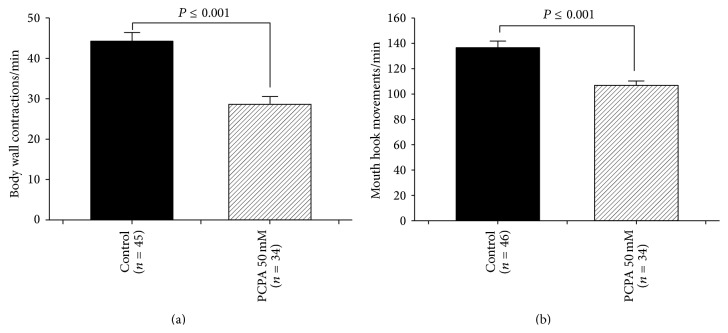
The behavioral consequences of blocking 5-HT synthesis. (a) Effect of tryptophan hydroxylase inhibitor,* para*-chlorophenylalanine (PCPA), on locomotion behavior. PCPA administration significantly decreased body wall contractions. (b) PCPA 50 mM significantly reduced feeding behavior. Student's *t*-test was performed to carry out comparison between groups. Data presented as mean ± SEM.

The rate-limiting enzyme of 5-HT synthesis was blocked by* para*-chlorophenylalanine (PCPA) administration. So, instead of increasing 5-HT, a decrease in levels in the CNS as well as systemic levels in 5-HT occurs. A similar feeding regime was used as in earlier studies which measured 5-HT with HPLC and showed a significant reduction in level of 5-HT [38]. The embryos were collected and were placed inside a mixture of 0.5 gm standard fly food and 0.5 mL water (control CS) or 0.5 gm food and 0.5 mL water plus PCPA. Feeding PCPA 50 mM significantly reduced the BWCs and MHMs (Figures 3(a) and 3(b), resp.).

### 3.4. Genetic Manipulation of Trh Expression

**Figure 4:**
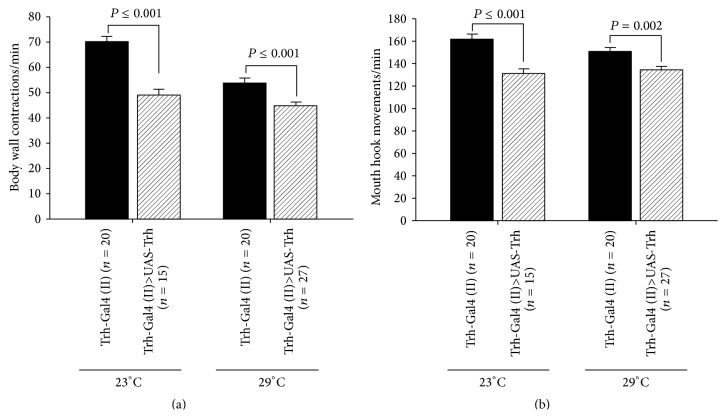
Overexpression of 5-HT biosynthesis rate-limiting enzyme, tryptophan hydroxylase (Trh), in serotonergic neurons. (a) Trh overexpression reduces body wall contractions. (b) Trh overexpression significantly decreased mouth hook movements as an indicator for feeding behavior. The flies were raised at 23°C as well as at 29°C to manipulate the expression level of transcriptional activator Gal4. Student's *t*-test was performed to carry out comparison between groups.

Tryptophan hydroxylase is the enzyme which promotes the synthesis of 5-HT. Thus, overexpression of* Trh* was predicted to result in similar alterations to feeding 5-HT or the 5-HTP.* Trh* was selectively overexpressed in serotonergic neurons for comparisons. The flies were raised at 23°C or 29°C to increase the expression level of Gal4 transcriptional activator. The proposed* Trh* overexpression significantly reduced the BWCs and MHMs in third-instar larvae (Figures 4(a) and 4(b), resp.). The 5-HT levels were not measured within the hemolymph for this strain; however, we assume the strain works as well as it has in previous studies. In addition, one of the parental lines (Trh-Gal4 (II)) was used as a control.

### 3.5. Serotonin Reuptake Blocker and Behaviors

**Figure 5:**
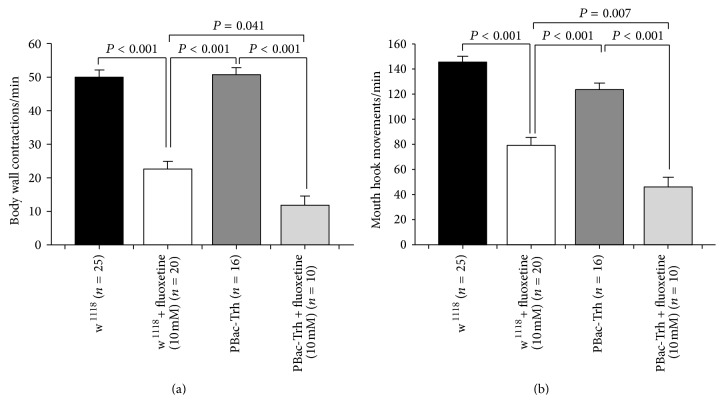
Serotonin reuptake inhibitor (SSRI), fluoxetine, influences both locomotion and feeding behaviors. (a) Fluoxetine significantly decreased body wall contractions. Interestingly, fluoxetine had a greater effect on tryptophan hydroxylase (Trh) mutant animals than w^1118^ animals. (b) Fluoxetine significantly reduced mouth hook movements. Fluoxetine, also, had a greater effect on tryptophan hydroxylase (Trh) insertional mutant (homozygous) animals than w^1118^ animals. One-way ANOVA was used for comparison, and Bonferroni *t*-test was employed to compare treatments. Data represented as mean ± SEM.

An additional approach to raise 5-HT at the synaptic cleft is to block the reuptake of 5-HT back into the presynaptic nerve terminal with selective 5-HT reuptake blockers. Fluoxetine was shown to block reuptake for the 5-HT transporter in* Drosophila* [27, 47]. Early second-instar larvae were collected and then 0.5 gm fly food was mixed with 0.5 mL water in case of control. Fluoxetine 10 mM was dissolved in 0.5 mL water, and then it was mixed with 0.5 gm food. After 48 hours, the behavioral assays were carried out. Since fluoxetine is light sensitive, therefore the larvae were placed in an incubator without light. Controls were treated similarly with light exposure. Fluoxetine 10 mM significantly reduced the BWCs and MHMs in w^1118^ larvae and Trh insertional mutant larvae compared to control groups (Figures 5(a) and 5(b)). Moreover, fluoxetine 10 mM in Trh insertional mutant larvae significantly reduced BWCs and MHMs in comparison to fluoxetine-fed w^1118^ larvae (Figures 5(a) and 5(b)).

### 3.6. Activation of 5-HT Neurons and Knockdown of 5-HT Receptors: Effects on Behavior

**Figure 6:**
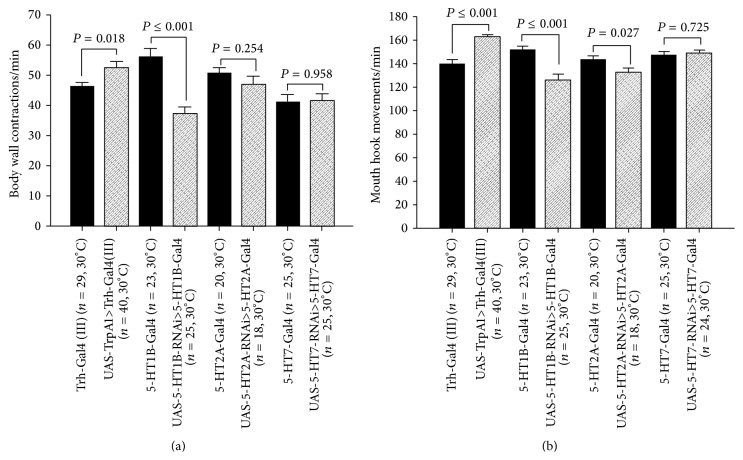
Manipulation of the 5-HTergic system. (a) Activation of serotonergic neurons, by using thermogenetic approach (TrpA1 channels), during early stages of development modulates locomotion behavior in later stage of development. RNA interference- (RNAi-) directed 5-HT1B receptor knockdown during early stage of development significantly reduced the locomotion behavior in third-instar larvae. (b) Activation of serotonergic neurons increased the feeding behavior. RNAi-directed 5-HT1B and 5-HT2A receptors significantly reduced the feeding behavior in third-instar larvae. Student's *t*-test was performed to carry out comparison between groups. Data presented as mean ± SEM.

Chronic activation of the 5-HT neurons through activating TrpA1 channels selectively expressed in 5-HT neurons significantly increased BWCs and MHMs (Figures 6(a) and 6(b)). 5-HT1B knockdown in 5-HT1B expressing neurons during development significantly reduced the BWCs and MHMs (Figures 6(a) and 6(b)). However, 5-HT2A and 5-HT7 receptor knockdowns in 5-HT2A and 5-HT7 receptor expressing neurons did not markedly influence the BWCs but did decrease MHMs (Figures 6(a) and 6(b)). All the flies were raised at 30°C to increase the Gal4 transcriptional activator activity. Similar results presented later in which the 5-HT containing neurons were stimulated with light, through expression of channel rhodopsin, complement these findings. In addition, the use of one of the parental strains (Gal4) as a control supports the differential findings for the F1 strains.

### 3.7. Manipulation of 5-HT Synthesis and Receptors: Effects on Behavior

**Figure 7:**
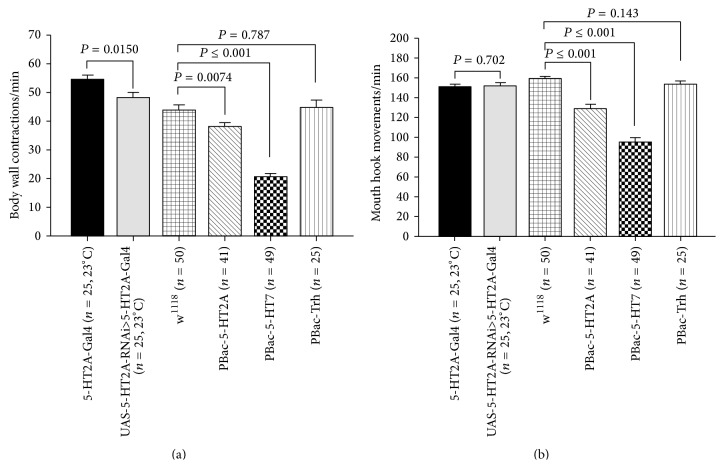
Serotonergic system dysregulation and behaviors. (a) Homozygous insertional mutations in 5-HT2A and 5-HT7 receptors significantly reduced body wall contractions in third-instar larvae. However, homozygous insertional mutation in 5-HT biosynthesis rate-limiting enzyme, tryptophan hydroxylase (Trh), did not have a significant effect on body wall contraction in comparison to w^1118^ group. (b) Homozygous insertional mutation in 5-HT2A and 5-HT7 receptors significantly reduced mouth hook movements in third-instar larvae. However, 5-HT2A receptor knockdown and homozygous Trh mutant larvae did not show noticeable effect on mouth hook movements. Student's *t*-test was performed to carry out comparison between groups. Data presented as mean ± SEM.

**Figure 8:**
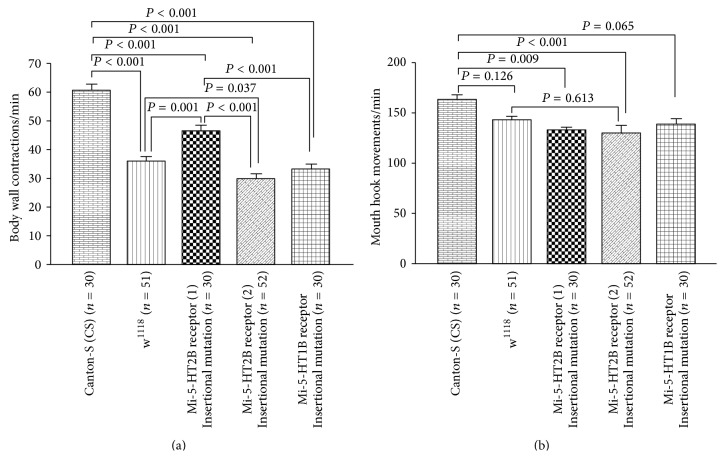
Manipulation in expression of mutated 5-HT receptors. (a) In all mutant lines, body wall contractions were significantly reduced in comparison to CS flies. However, 5-HT2B receptor (line 2) insertional mutant larvae significantly reduced body wall contractions in comparison to w^1118^ larvae. (b) Mouth hook movements were significantly affected in 5-HT2B receptor mutations in comparison to CS larvae, although mouth hook movements in 5-HT2B receptor (line 2) insertional mutant larvae were not significantly reduced compared to w^1118^ larvae. One-way ANOVA was used for comparison, and Bonferroni *t*-test was employed to compare treatments. Data represented as mean ± SEM. *P* value < 0.05 is significant.

In determining which receptor subtypes may play a larger role in altering the larval behaviors, strains carrying insertional mutations in 5-HT receptors were examined. In addition, a PBac-Trh strain was examined which would perturb the synthesis of 5-HT. The insertional mutation in 5-HT2A (homozygotes mutant) and 5-HT7 (homozygotes mutant) receptors significantly reduced the BWCs and MHMs in comparison to w^1118^ larvae (Figures 7(a) and 7(b)); however, Trh mutant larvae did not show a noticeable change in BWCs and MHMs compared to w^1118^ larvae (Figures 7(a) and 7(b)). Figures 8(a) and 8(b) show that the fly lines were significantly reduced in BWCs and MHMs in comparison to control CS larvae. Furthermore, 5-HT2B mutant (line 2) larvae significantly decreased BWCs in comparison to w^1118^ larvae, although there was not a marked change in MHMs between w^1118^ and 5-HT2B mutant (line 2) larvae (Figures 8(a) and 8(b)). The 5-HT2B mutant (line 2) had a larger decrease in BWC (Figure 8(b)) than 5-HT2B mutant (line 2). However, there was no difference between these strains in MHMs.

### 3.8. Manipulation of Synaptic Activity in 5-HT Neurons within Larvae

**Figure 9:**
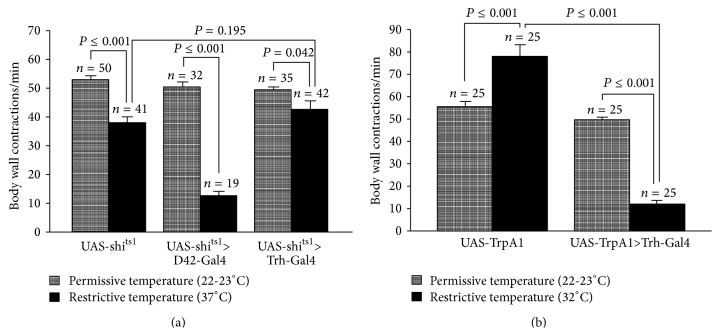
Acute manipulation of 5-HTergic neurons activity by* shi* ^*ts1*^ and TrpA1. (a) Synaptic transmission was blocked in the 5-HTergic neurons by expressing dominant negative temperature sensitive* shibire* ^*ts1*^ allele (*shi* ^*ts1*^). Suppression of synaptic transmission in motor neurons (UAS-*shi* ^*ts1*^>D42-Gal4) significantly reduced body wall contractions; however, suppression of synaptic transmission in 5-HT neurons (UAS-*shi* ^*ts1*^>Trh-Gal4 (III)) did not significantly influence body wall contractions. The flies were raised at 23°C, 12 : 12 LD cycle. To obtain the locomotion behavior at room temperature for control and* shibire* ^*ts1*^ expressing larvae, an individual third-instar larva was placed on the apple-juice agar. The larva was left for 1 min to acclimate, and body wall contractions were counted for the following 1 min. To obtain body wall contractions at restrictive temperature for control and* shibire* ^*ts1*^ expressing larvae, the larvae were incubated at 37°C for 20 min. The individual larvae were placed on prewarmed apple-juice agar on hot plate (30–32°C). The larva was left for 1 min to acclimate, and the body wall contractions were counted for the following 1 min. (b) Thermogenetic approach was used to activate 5-HTergic neurons. When TrpA1 channels are exposed to high temperature (32°C), they become activated which leads to inward current flux of cations. Activation of 5-HTergic neurons (UAS-TrpA1>Trh-Gal4 (III)) significantly decreases BWCs compared to control larvae (UAS-TrpA1). To obtain BWCs at room temperature, larvae were placed on apple-juice agar dish at room temperature and left for 2 min to acclimate. Afterwards, BWCs were counted for 1 min. The locomotor behavior at restrictive temperature (32°C) was obtained by placing one larva on a prewarmed apple-juice agar dish on a hot plate (32°C). The larvae were left for 2 min, and then the BWCs were counted for 1 min. Student's *t*-test was performed to carry out comparison between groups. Data presented as mean ± SEM. *P* < 0.05 is significant.

The dominant negative allele* shi* ^*ts1*^ was expressed in serotonergic neurons to block synaptic transmission.* shi* ^*ts1*^ is temperature sensitive which functions well at permissive temperature (22-23°C); however, it does not function at restrictive temperature (37°C). Thus, the synaptic vesicles within the presynaptic nerve terminals are not able to recycle. So there is an overall reduction in 5-HT being released in evoked and spontaneous vesicular events. The flies were raised at room temperature, and the third-instar larvae were selected and incubated at 37°C for 20 min. It was shown that expressing* shi* ^*ts1*^ in sensory neurons significantly reduces locomotion behavior when they are incubated at 37°C for 20 min [39]. In this study, larvae which were expressing* shi* ^*ts1*^ in 5-HT neurons did not markedly alter the rate in the body wall contractions compared to control (UAS-*shi* ^*ts1*^) (Figure 9(a)). The activity of 5-HT neurons was increased by expressing temperature sensitive channel, TrpA1. It has been demonstrated that TrpA1 does not become activated at room temperature (22-23°C) but when it is exposed to >28°C, the TrpA1 channels become activated [41]. The larvae that were expressing TrpA1 channels were put on a prewarmed (32°C) agar plate while the body wall contractions were being counted. The data indicate that acute activation of 5-HT neurons in third-instar larvae significantly decreased BWCs at 32°C compared to control group (UAS-TrpA1) (Figure 9(b)). One of the parental lines (UAS-TrpA1) was used for comparisons. The locomotion activity in third-instar larvae was also decreased when 5-HT neurons were activated by light, through expression of channelrhodopsin-2 (ChR2), which complements these findings (see the optogenetics results).

### 3.9. Manipulation of Activity in 5-HT Neurons within Adults

**Figure 10:**
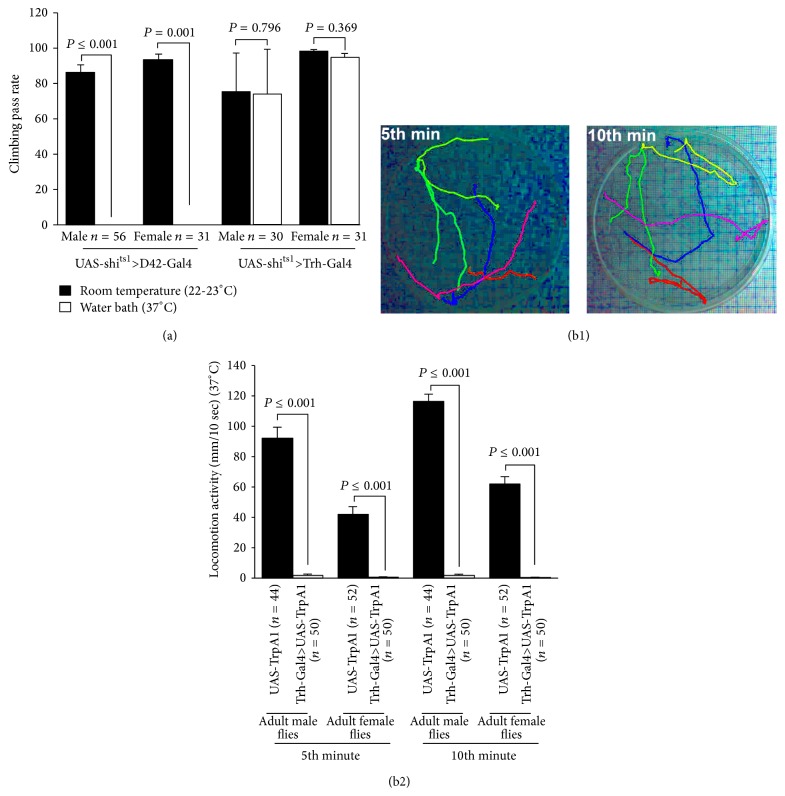
Manipulation of activity in 5-HTergic neurons within adult flies. (a) Acute inhibition of neuromuscular synapse neurotransmission by expressing* shi* ^*ts1*^ in motor neurons (UAS-*shi* ^*ts1*^>D42-Gal4) significantly reduced climbing ability in adult female and male flies at restrictive temperature (37°C). This assay served as a proof of concept in the genetic crosses. However, acute blockage of serotonergic synaptic transmission (UAS-*shi* ^*ts1*^>Trh-Gal4 (III)) did not influence climbing ability in adult flies. Student's *t*-test was used to compare the climbing ability of the flies at room temperature (22-23°C) with the same flies after incubation in the water bath (37°C). (b1) Locomotion activity was recorded for 10 minutes in UAS-TrpA1 (control) and Trh-Gal4 (III)>UAS-TrpA1, which expressed TrpA1 in 5-HTergic neurons, flies at 37°C. The activity was analyzed for 10 seconds at the 5th minute and 10 seconds at the 10th minute. These two pictures show the locomotion activity of adult male flies (controls), 10 sec of 5th and 10 sec of 10th minute, respectively. (b2) Acute activation of 5-HTergic neurons in freely moving adults significantly reduced the locomotion activity in adult male and female flies. Mann-Whitney rank sum test was used for comparison between the groups. Data represented as mean ± SEM.

Flies were raised at room temperature (22-23°C), which were expressing a temperature sensitive dominant negative* shi* ^*ts1*^ allele in 5-HT neurons. The climbing assay (i.e., negative geotaxic assay) was carried out to observe the effect of acute activity in 5-HT neurons on locomotion behavior in adult flies. The* shi* ^*ts1*^ flies were crossed with motor neuron specific Gal4 (D42-Gal4) and this cross was used as a positive control. When the flies were placed at 37°C for 10 min, locomotion activity significantly decreased. Furthermore,* shi* ^*ts1*^ flies were crossed with 5-HT neurons specific Gal4 driver (Trh-Gal4 (III)). When these flies were incubated at 37°C for 10 min in the water bath, no significant changes were observed in locomotion behavior in both males and females in comparison to control flies (Figure 10(a)). The climbing assay, which was performed in* shi* ^*ts1*^ fly groups, was not a suitable assay for the locomotion behavior of TrpA1 expressing flies since when they were placed at 37°C, the control flies were very active but they stayed at the bottom while the climbing assay was being performed (i.e., they did not cross the 8 cm line at the 10 sec time period); therefore, we decided to use another assay to record locomotion activity as depicted in Figure 10(b1). Adult flies (4-5 flies in each group, males and females separately) were placed in a Petri dish which allowed the adults to walk around horizontally but not fly. Both controls (UAS-TrpA1) and TrpA1 expressing flies were transferred to a 37°C room. The locomotor activity was recorded for 10 min, and the activity was analyzed for 10 sec at 5th and 10 sec at 10th min. Acute activation of TrpA1 channels in 5-HT neurons significantly reduced the locomotor activity in male and female TrpA1 expressing flies at 5th and 10th min compared to male and female control groups (UAS-TrpA1). Note that the females had a decreased locomotor activity compared to male flies (Figure 10(b2)).

The locomotor activity in adult flies was also decreased when 5-HT neurons were activated by the channelrhodopsin ChR2-XXL (Figure 17(c)). These results complement the results obtained from TrpA1 experiments. The other less sensitive ChR2 variants were not used in this experiment since the adult flies have a thick cuticle which reduces the level of blue light penetrance; therefore, blue light cannot activate enough ChR2 to observe a change in the behavior. Also, we showed that blue light exposure is effective in flies that were not fed ATR. It has been shown previously that ChR2-XXL can be activated in the absence of ATR diet supplementation [28].

### 3.10. Sensory-Motor Circuit Activity and 5-HT

Sensory-motor circuit activity was used to examine the influence of 5-HT on the circuit activity in dissected third-instar larvae. The rationale of conducting these acute studies in dissected preparations is to directly examine the action of 5-HT on a neural circuit in which one could control the electrical activity of sensory neurons and examine a defined motor output on a body wall muscle. The body wall muscle (m6) is used in the intact larvae for movements measured in BWM behavioral assay. Therefore, application of compounds which alter synaptic 5-HT in the excised preparation can be correlated to the approaches used to manipulate 5-HT concentration through diet, pharmacology, and genetics.

**Figure 11:**
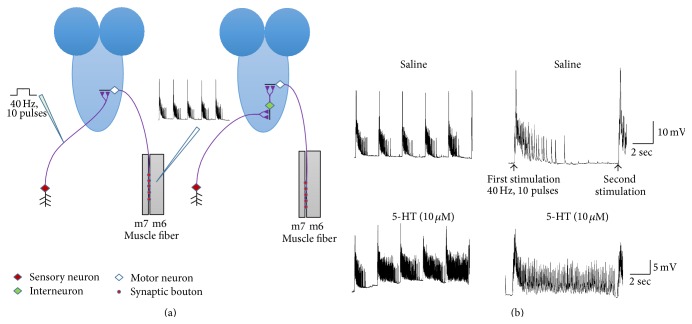
The effect of 5-HT on a CNS circuit. (a) shows two hypothetical models for the sensory-motor circuit. The first model is a monosynaptic model (left side) with sensory neurons directly making synaptic connections with motor neurons. The second model is a multisynaptic one in which the sensory neurons make synaptic connections with interneurons and then the interneurons make connections with the motor neurons. The two segmental nerves that innervate the last segment were cut and pulled into a suction electrode. The segmental nerves were stimulated at 40 Hz, 10 pulses, and the excitatory postsynaptic potentials (EPSPs) were being recorded from body wall muscle fiber 6 or 7. (b) The modulatory action of 5-HT on sensory-motor function in third-instar larvae. Application of 5-HT noticeably increased the frequency of evoked EPSPs. The far right traces are enlarged depictions of one evoked burst shown to the left.

Sensory neurons of the last segmental nerves in early 3rd instars were stimulated at 40 Hz, 10 pulses (Figure 11(a)). The segmental nerves were stimulated for 8 stimulation trains in the saline and 8 stimulation trains or more after exchanging saline with saline containing 5-HT, 5-HT agonists, a selective serotonin reuptake inhibitor (SSRI, fluoxetine), or prewarmed saline, which was used in TrpA1 expressing larvae. When saline was exchanged with saline-containing 5-HT, the number of evoked excitatory postsynaptic potentials (EPSPs) significantly increased (Figure 11(b)).

### 3.11. Pharmacological Study of 5-HT Receptor Subtypes

**Figure 12:**
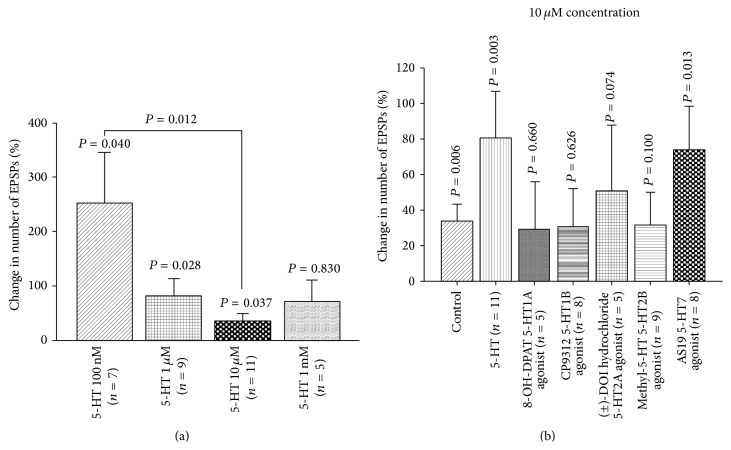
5-HT and sensory-motor function modulation in third-instar larvae. (a) Dose-response relationship of 5-HT in sensory-motor physiology. 5-HT significantly increased the frequency of EPSPs. The EPSPs of 8 stimulations were recorded for each saline and after exchanging saline with various 5-HT concentrations. (b) Effect of various 5-HT receptor agonists on the frequency of EPSPs in third-instar larvae. Application of 5-HT and 5-HT7 agonist significantly increased the number of EPSPs. The segmental nerve was stimulated at 40 Hz, 10 pulses, and the EPSPs were recorded from muscle fiber 6 or 7. Seven to ten stimulations were analyzed in each saline and after exchanging saline with a saline that contained agonist or in case of the controls fresh saline was used. Paired *t*-test was used to compare the number of EPSPs in saline and saline that contained an agonist. Data represented as mean ± SEM. *P* < 0.05 is significant.

Application of 5-HT at 100 nM to the exposed larval brains significantly increased the number of EPSPs. When higher concentrations of 5-HT (1 *μ*M, 10 *μ*M) were applied, the number of EPSPs was also significantly increased; however, the percentage change at 100 nM was higher than the percentage change for higher concentrations (Figure 12(a)). Various 5-HT agonists were used to observe their actions on the sensorimotor activity. This demonstrates that 5-HT7 agonist, AS19, and 5-HT2 agonist (±)-DOI HCl noticeably increased the number of EPSPs; however, no significant changes were observed after application of 8-OH-DPAT, CP9312, and *α*-methyl-5-HT (Figure 12(b)). There is one study which demonstrated that DOI appears to function at 5-HT2 receptors in* Drosophila* [48].

**Figure 13:**
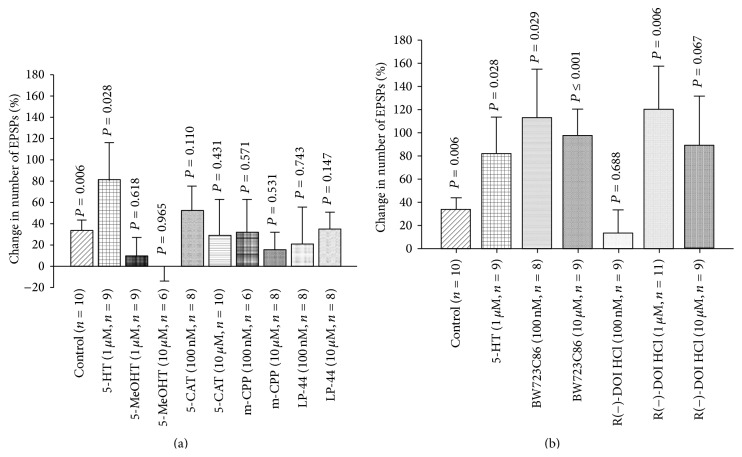
Modulation of motor-sensory function by 5-HT agonists. (a) Effect of various 5-HT agonists on the number of EPSPs in third-instar larvae. The control group was obtained by changing saline with fresh saline to observe the effect of the saline switching process on EPSPs frequency. The results show that 5-HT significantly increased the number of EPSPs; however, 5-methoxytryptamine (5-MeOHT), 5-carboxamidotryptamine maleate salt (5-CAT), 1-(3-chlorophenyl)piperazine hydrochloride (m-CPP), and LP-44 (5-HT7 agonist) did not produce significant changes. (b) 5-HT2 agonists, BW723C86 (100 nM and 10 *μ*M) (5-HT2B receptor agonist) and R(−)-DOI HCl (1 *μ*M) (5-HT2 receptor agonist), significantly increased the motor-sensory circuit activity. Paired *t*-test was used to compare the number of EPSPs in saline with saline that contained an agonist. Data represented as mean ± SEM. *P* < 0.05 is significant.

Other 5-HT agonists were used for further confirmation of the 5-HT receptors subtypes that mediate modulatory role of 5-HT in sensory-motor circuit activity. 5-MeOHT (1 *μ*M and 10 *μ*M), 5-CAT (100 nM and 10 *μ*M), m-CPP (100 nM and 10 *μ*M), and LP-44 (100 nM and 10 *μ*M) were applied to dissected third-instar larvae. There were no significant changes in EPSPs after application of these agonists (Figure 13(a)). Moreover, different 5-HT2 agonists were employed for further confirmation. 5-HT2 agonists (BW723C86) (100 nM and 10 *μ*M) and R(−)-DOI HCl (1 *μ*M) significantly increased the number of EPSPs (Figure 13(b)). As far as we know, only R(−)-DOI HCl was examined for binding to* Drosophila* 5-HT receptors; however, to the best of our knowledge, binding studies of BW723C86 have yet to be performed on* Drosophila* specific 5-HT receptors.

### 3.12. Genetic Study of 5-HT Receptor Subtypes

**Figure 14:**
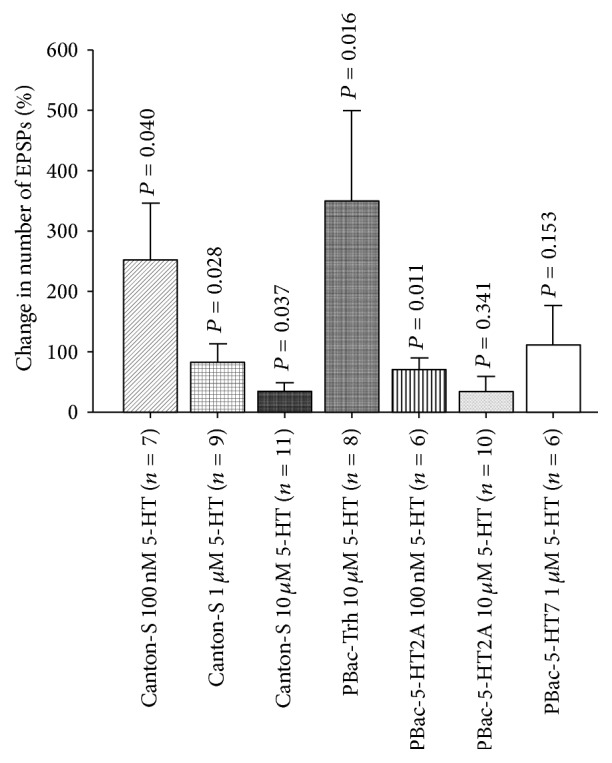
Effect in sensitivity to 5-HT modulation in line with disrupted aspects within the 5-HTergic system. 5-HT application on tryptophan hydroxylase (Trh) mutant third-instar larvae markedly increased sensory-motor circuit physiology. 5-HT at 10 *μ*M exposure in CS third-instar larvae seems to desensitize 5-HT receptors since it does not markedly modulate neural circuitry in comparison to 5-HT 100 nM. 5-HT2A receptor mutation significantly reduced the modulatory action of 5-HT. However, mutation in 5-HT7 receptor noticeably responds to 5-HT at 1 *μ*M. Paired *t*-test was used to compare the number of EPSPs in saline with saline that contained 5-HT. Data represented as mean ± SEM. *P* < 0.05 is significant.

5-HT receptor subtype mutant lines mentioned previously were used for electrophysiological studies to corroborate the pharmacological results. 5-HT significantly increased the number of EPSPs in Trh mutant larvae. Also, the response of Trh mutant larvae to 5-HT was higher than control flies (CS) (Figure 14). In addition, the exogenous application of 5-HT in dissected third-instar larvae significantly increased the sensorimotor activity in 5-HT2A receptor mutant flies, although the response of 5-HT2A receptor flies to 5-HT was lower than the control flies (CS). In 5-HT7 mutant larvae, 5-HT application slightly increased the sensory-motor activity but at *P* > 0.1 for group comparisons from controls due to the high variation in this line (Figure 14).

### 3.13. Neural Circuit Activity and Acute 5-HTergic Neurons Activation

**Figure 15:**
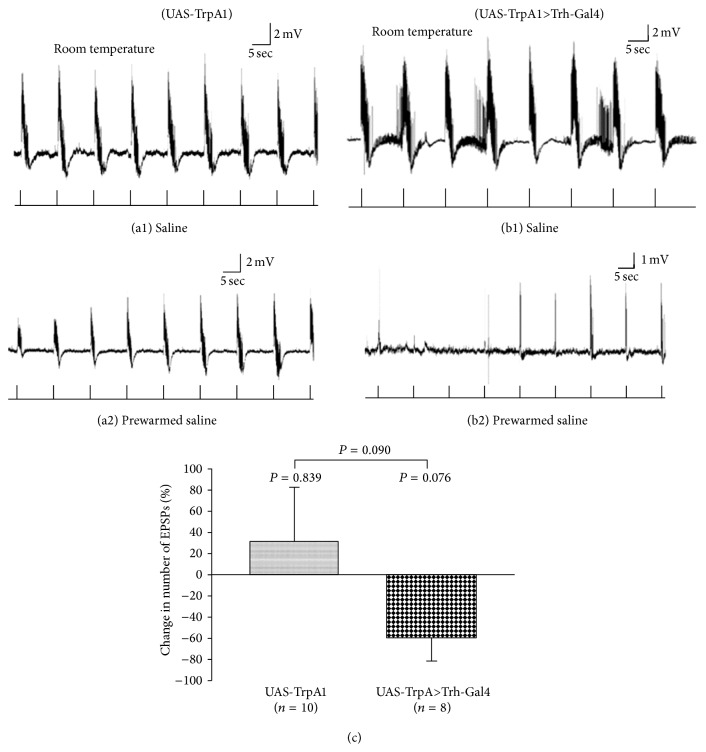
Induced activity in 5-HTergic neurons and sensory-motor physiology in third-instar larvae. (a1) The segmental nerves of dissected control (UAS-TrpA1) third-instar larvae were stimulated at 40 Hz, 10 pulses, in saline at room temperature. (a2) The saline was exchanged with prewarmed saline in a water bath (37°C) and then applied to the preparation at 32–34°C. (b1) 5-HTergic neurons expressing temperature sensitive cation channel, TrpA1 (Trh-Gal4 (III)>UAS-TrpA1). The evoked activity (EPSPs) of sensory-motor neural circuit was recorded at room temperature (22-23°C) saline. (b2) Saline was switched with prewarmed saline in a water bath (37°C) when the evoked activity was being recorded. Activation of 5-HTergic neurons significantly reduced evoked neural activity. The prewarmed saline was transferred to the dissection dish for both control and TrpA1 expressing third-instar larvae. (c) Activation of 5-HT neurons markedly reduced the number of EPSPs compared to control larvae. Paired *t*-test was used to compare the frequency of EPSPs inside room temperature saline and prewarmed saline. Student's *t*-test was used to compare different treatments. Data represented as mean ± SEM.

To examine overactivity of the 5-HTergic neurons, the same sensory-CNS-motor paradigm was used in third-instar larvae (UAS-TrpA1) at room temperature (22-23°C) (Figure 15(a1)) and when switched with prewarmed saline (37°C). When the saline was applied to the preparation it was 32–34°C.

**Figure 16:**
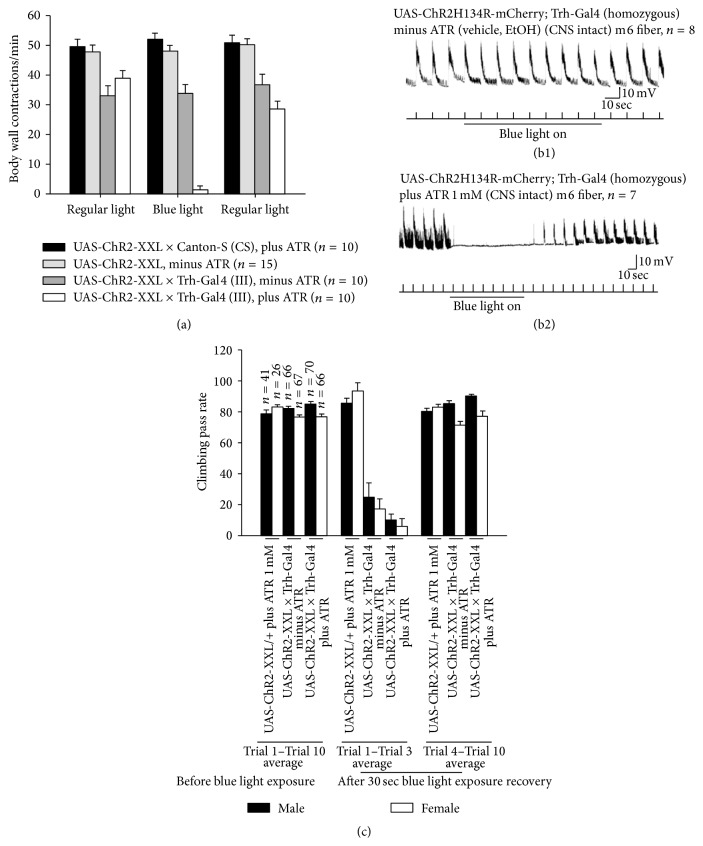
Serotonergic neuron activation modulates locomotor behavior in third-instar larvae and adult flies. (a) To change serotonergic neuron activity, ChR2 was expressed in serotonergic neurons (UAS-ChR2-XXL/+; Trh-Gal4/+). The body wall contractions were counted in third-instar larvae fed on food supplemented with ATR 1 mM or ethanol (vehicle). When the larvae, which were fed on ATR 1 mM, were exposed to blue light, the locomotor activity was significantly compromised. However, when the larvae fed on a food without ATR (b1) supplementation were exposed to blue light, the locomotor activity was not significantly affected. Fly food not supplemented with ATR and exposed to blue light did not suppress sensorimotor activity in dissected third-instar larvae; however, (b2) serotonergic neuron activation by exposing the third-instar larvae, which were fed food supplemented with ATR (1 mM), to blue light shut down sensory-motor circuit activity. The sensory-motor circuit was being activated by trains of stimulations at 40 Hz, 10 pulses, while the motor output was being recorded in abdominal muscle 6 (m6). (c) Climbing assay in adult flies was used to test the locomotive ability of the flies. Activation of serotonergic neurons in adult flies decreases climbing ability. The excitability of serotonergic neurons is increased by expressing and activating ChR2 by blue light. When the adult flies were being exposed to blue light, the climbing ability significantly reduced. Both flies groups (UAS-ChR2-XXL/+; Trh-Gal4/+) which were fed food supplemented with ATR 1 mM or ethanol (vehicle) were affected by the blue light exposure. However, blue light did not have effect on the control lines (UAS-ChR2-XXL × Canton-S, shown as UAS-ChR2-XXL/+). The climbing assay was repeated 10 times and the average (Trial 1–Trial 10 average) was taken. There was a 1 min rest interval between two trials before blue light exposure. Moreover, after exposing the flies to blue light for 30 sec, the climbing assay was repeated for 10 trials. The average was taken for Trial 1–Trial 3 since the locomotion activity was decreased during this period. The average was taken for Trial 4–Trial 10, in which the flies started recovering after blue light exposure.

The numbers of EPSPs of 7-8 stimulation trains bathed in saline and 7-8 stimulation trains inside prewarmed saline were counted (Figures 15(a1) and 15(a2)). 5-HTergic neurons were activated while the motor output was being recorded from muscle fiber 6 or 7 in TrpA1 expressing larvae in saline (Figure 15(b1)) and warmed saline (Figure 15(b2)). The results indicate that acute activation of 5-HTergic neurons by TrpA1 cation channels (Trh-Gal4 (III)>UAS-TrpA1) showed a decrease in activity when warmed at 92.4% significance (*P* = 0.076) and in comparing this line to the changes in the parental line (UAS-TrpA1) only a change occurred at 91% significance (*P* < 0.09) (Figure 15(c)). We choose to use another approach which has recently become popular due to the specific activation of known neurons. We used the optogenetic approach to selectively activate 5-HTergic neurons while the sensorimotor neuron activity was being recorded. In dissected third-instar larvae, blue light exposure did not suppress sensorimotor activity in UASChR2H134R-mCherry; Trh-Gal4 (homozygous for both constructs) larvae which were not fed ATR (Figure 16(b1)). But blue light exposure suppressed sensorimotor activity in UAS-ChR2H134R-mCherry; Trh-Gal4 (homozygous for both constructs) larvae which were fed food supplemented with ATR (1 mM) (Figure 16(b2)).

### 3.14. Fluoxetine and Sensory-Motor Activity

**Figure 17:**
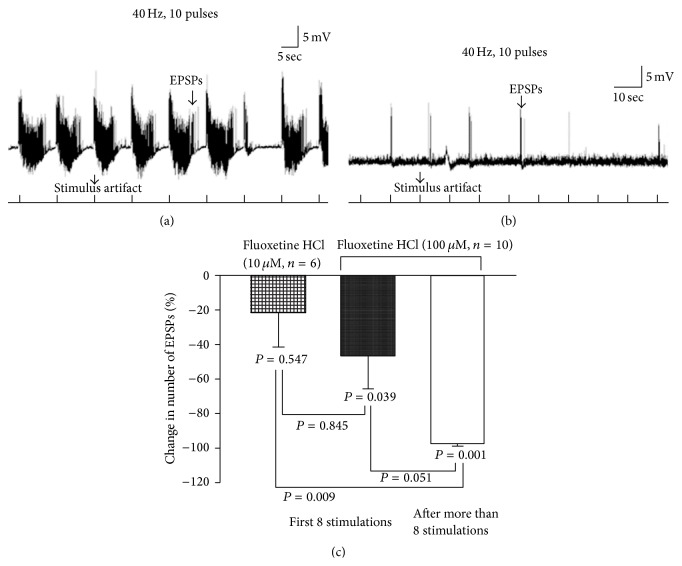
Motor-sensory function and action of a selective serotonin reuptake inhibitor (SSRI). (a) Evoked sensory-motor neural activity (EPSPs) was recorded from body wall muscle fiber 6 or 7 of third-instar larvae in saline. (b) Application of fluoxetine (100 *μ*M) suppresses the evoked activity of the neural circuitry. (c) Fluoxetine at 10 *μ*M did not significantly decrease activity but there is a decreasing trend in the mean change in the sensory-motor function. Paired *t*-test was used to compare the frequency of EPSPs inside saline and fluoxetine. One-way ANOVA was used to compare different treatments. Bonferroni *t*-test was employed to obtain significant results. Data represented as mean ± SEM. *P* < 0.05 is significant.

The sensory-motor circuit was stimulated at 40 Hz, 10 stimuli, and the EPSPs were recorded in saline (control) (Figure 17(a)). Afterwards, fluoxetine 10 *μ*M or 100 *μ*M was applied to observe how it influenced the sensorimotor circuit physiology. Fluoxetine is a selective serotonin reuptake inhibitor (SSRI) which would potentially cause a rise in the 5-HT within the synaptic cleft. Application of fluoxetine 100 *μ*M significantly reduced the number of EPSPs (Figure 17(b)). After 10 min incubation time, fluoxetine nearly completely suppressed the sensorimotor activity. However, no significant reduction was observed when fluoxetine 10 *μ*M was applied to dissected third-instar larvae (Figure 17(c)).

## 4. Discussion

In the current study, we investigated the role of the 5-HTergic system in locomotion and feeding behaviors as well as activity in a sensorimotor circuit. 5-HT alone can enhance the evoked activity; however, 5-HT likely interacts with other known neuromodulators such as dopamine and octopamine, to fulfill specific physiological or behavioral functions [49]. It has been demonstrated that dysregulation in the 5-HTergic system is related to various psychological conditions such as depression, anxiety, learning and memory impairment, schizophrenia, and autism in humans [50]. The disruption of physiological serotonin level in critical periods during mammalian development is postulated to abnormal development of neural circuitry that consequently results in the long-lasting psychological disorders [51]. Many studies have already shown that 5-HT plays miscellaneous physiological and behavioral roles in* Drosophila*. Also, it is known that 5-HT level plays a role in development of the 5-HTergic neurons in the central nervous system (CNS) of* Drosophila* [52]. A previous study has demonstrated that 5-HT2 receptors are expressed in* Drosophila* during the embryonic stage [48], and its physiological level of expression is essential for the normal development and survival [53]. However, the physiological role of 5-HT receptor subtypes in various neural circuit activities had yet to be further investigated.

### 4.1. 5-HT Action and Locomotion Behavior

Neuromodulators, such as dopamine and serotonin, play a role in changing the activity of locomotor neural networks [54]. Many studies have shown that 5-HT is implicated in locomotion behavior in diverse animals and relatively simple organisms such as* C. elegans* [32, 55]. It has been shown that the sensory input is indispensable for the locomotion activity of* Drosophila* larvae [39]. The rapid central drive in locomotion is rapidly altered when an organism confronts eminent danger, which may well be influenced by neuromodulators. Neuromodulators are found in* Drosophila* which function to modulate specific behaviors [49] and thus maybe even survival in the outdoor environment under predatory stress. It was also shown recently that various 5-HT receptors have a role in* Drosophila* larvae locomotion [6] and that 5-HT is involved in larval turning behavior [56]. In the study herein, the 5-HT system was manipulated by using both pharmacological and genetic approaches and complements some aspects of the results reported by Silva et al. [6]. We showed that feeding larvae high levels of 5-HT or 5-HTP significantly reduced body wall contractions and reduced the body length. It is likely that 5-HTP also led to an increase in 5-HT synthesis but this does not necessarily mean more 5-HT is released with synaptic transmission. It was shown that administration of 5-HTP increases the level of 5-HT in adult flies [57]. 5-HT could alter* Drosophila* development by acting on insulin producing cells (IPCs) in the brain [10, 58–60]. This could prepare the organism for increased metabolic activity which can also be induced by promoting locomotive activity. The mechanism for the retarded growth with very high levels of 5-HT is only speculative at this time. Additionally, the Trh blocker, PCPA, significantly reduced locomotion behavior and it was demonstrated that PCPA administration results in the reduction of 5-HT in larval CNS [38]. These results demonstrate that dysregulation of 5-HT with either too much or too little 5-HT influences locomotion behavior. We further investigated the effect of 5-HT biosynthesis disturbance on locomotive behavior by overexpression of the Trh gene specifically in 5-HTergic neurons which also slowed larval locomotion.

In order to determine whether 5-HT was increased in the synaptic cleft of 5-HT releasing neurons, we used an SSRI (fluoxetine). Such potential increase in synaptic 5-HT also decreased locomotion behavior. The effect was even more substantial in the Trh mutant larvae. It has been confirmed that, in Trh mutant larvae, the 5-HT level is reduced in 5-HT neurons. Possible residual 5-HT in neurons might be due to the 5-HT uptake by dSERT from the hemolymph, which is made by the peripheral DTHPu. This may explain why administration of fluoxetine (5 mg/mL) further reduces 5-HT level in Trh mutant 5-HT neurons [61]. The action of fluoxetine may indeed have other effects and side effects in invertebrates. The affinity of fluoxetine for* Drosophila* SERT was previously determined to be lower (*K*_i_ = 72 nM) than for mammals [47]. High concentrations of fluoxetine are toxic to larval and adult* Drosophila* [62] so we stuck to a 10 mM concentration for this study.

### 4.2. Genetic Manipulation of 5-HTergic System

Increasing the activity of 5-HT neurons through activation of the TrpA1 channels selectively targeted these neurons and increased the locomotion behavior in third-instar larvae. This is in contrast to the other manipulations in the levels of 5-HT which all decreased locomotion. Potentially over a long period of heightened neural activity there might be some compensation by possibly downregulating the expression of 5-HT receptors or possibly desensitization of the receptors. Since we do not know if these neurons may have fatigued in the ATP production required for vesicular fusion and recycling or possibly depleted the release of 5-HT with the electrical activity, more definitive studies are needed to answer these questions. Further investigations with periodic heat pulses would be interesting to pursue. Various 5-HT receptor subtypes (5-HT1A, 5-HT1B, 5-HT2A, 5-HT7 [30], and 5-HT2B [9]) are expressed in* Drosophila*; thus, our approach with RNAi in 5-HT1B knockdown, in 5-HT1B expressing neurons, could specifically examine this receptor role in locomotion. It is interesting to note that the 5-HT1B knockdown significantly reduced the locomotion but 5-HT2A and 5-HT7 receptor knockdown did not have an effect. It is known that 5-HT acts on serotonergic neurons through activation of 5-HT autoreceptors to orchestrate the serotonergic neuron branch density [52, 63]. Thus, the knockdown of 5-HT1B during early stages of development might increase the 5-HT neurons branching pattern which might result in increasing 5-HT concentration thereby changing the development of the neural circuitry. This is another interesting avenue to follow up in the possible anatomical restructuring.

In a recent study, it has been shown that 5-HT receptor subtypes (5-HT1A, 5-HT1B, 5-HT2, or 5-HT7) knockdown in the nervous system increases locomotion activity when the flies were raised at 19°C until the day before examining the behavior [6]. However, in our study, we raised the flies at 30°C during early development. The paradoxical results might be due to the effect of 5-HT1B on larval development since the Gal4 activity would be high at 30°C compared to 19°C [64]. In addition, the higher temperature might increase the efficiency of the knockdown. The efficiency of the 5-HT2 and 5-HT7 RNAi lines to knock down 5-HT2 and 5-HT7 could be low since we did not observe a significant change in locomotion behavior. 5-HT receptors insertional mutant lines were also used and the results indicate that 5-HT2A and 5-HT7 insertional mutations significantly decreased locomotion behavior. However, Trh mutant larvae did not show a significant change in body wall contractions. Our results are consistent with Silva et al. [6] which demonstrated that 5-HT7 mutation leads to the reduction in locomotion activity; however, they were contradictory to Silva et al. [6] where the Trh mutation resulted in an increase in locomotive activity. In support of our findings for the Trh mutant, the results are consistent with Neckameyer et al. [61], where they demonstrated no significant difference between w^1118^ and PBac-Trh larvae body wall contractions. The contradictory results might be due to using different approaches to measure the locomotion activity in various studies. We wanted to address the effect of Trh overexpression on larval locomotion and feeding behaviors as well since previous studies have shown that Trh overexpression, which increases the 5-HT level, would change various fly behaviors such as sleep [17] and aggression [57]. We observed that Trh overexpression significantly reduced locomotion and feeding behaviors (Figures 4(a) and 4(b)), which corroborates the pharmacological results (Figure 2). In the study herein, the 5-HT2B receptor mutation, also, decreased body wall contractions in third-instar larvae (Figure 8(a)). Further support of 5-HT receptors being altered in their expression levels and the notion that the PBac-Trh larvae decreased 5-HT synthesis which could lead to upregulation of the receptors is that these larvae show an enhanced response to exogenous application of 5-HT on the exposed sensory-CNS-motor circuit. Together, these results demonstrate that dysfunction of 5-HT receptors during development negatively influences locomotion behavior. Additionally, we selectively activated 5-HT neurons with the use of optogenetics which demonstrated an effect on the sensory-CNS-motor circuit.

### 4.3. Acute Activation of 5-HTergic Neurons and Behavioral Modulation

Since an alteration in the 5-HTergic system during development might have effect on neural circuit development, it is difficult to pinpoint specific mechanisms to account for the behavioral changes. Therefore, we also used genetic tools to acutely manipulate 5-HT neurons in freely moving animals. The synaptic transmission of 5-HT neurons was blocked by expressing* shi* ^*ts1*^ allele in 5-HTergic neurons. Blockage of 5-HT neurons did not have an acute effect on the locomotion activity. However, dumping of 5-HT by increasing the activity of the 5-HTergic neurons, through TrpA1 channel activation, significantly reduced the body wall contractions in larvae, which corroborates the recently published findings of Pooryasin and Fiala [14]. This could suggest that 5-HT spillover from 5-HT neurons in freely moving larvae has a negative effect on locomotion behavior. In a recent study, it was stated that activation of TrpA1 channels negatively impacts locomotion activity in* Drosophila* larvae [56]. Furthermore, our results also show that suppression of synaptic transmission in 5-HT neurons did not cause a significant change in climbing behavior (Figure 10(a)); however, acute activation of 5-HT by TrpA1 significantly reduced the locomotion activities in adult male and female flies (Figures 10(b1) and 10(b2)). It was previously shown that adult* Drosophila* expressing* shi* ^*ts1*^ allele in 5-HT neurons and control group would increase the locomotion activity when they are exposed to high temperature (30°C) compared to low temperature (19°C). This effect is related to the temperature change and not blocking the transmitter release by the neurons [65].

Since the temperature likely has an effect on* Drosophila* behavior per se, we used an optogenetic approach to activate 5-HTergic neurons in freely moving larvae and adults. Our results show that activation of 5-HTergic neurons reduces locomotion behavior in third-instar larvae and adult flies (Figure 16).

### 4.4. Modulatory Role of 5-HT in Feeding Behavior

The 5-HTergic system, also, plays a role in feeding behavior in invertebrates [7, 44] and vertebrates [35]. For most parts, increasing or decreasing 5-HT altered the feeding behavior. In a previous study, it was demonstrated that 5-HTP administration decreases feeding behavior in larvae; however, reduction of 5-HT level through RNAi-mediated Trh knockdown increases feeding behavior. On the other hand, increasing Trh level decreases feeding. Also, the mutation in Trh gene increases the branching pattern of serotonergic neurons in proventriculus of the digestive system [13]. In our study, chronic activation of 5-HT neurons by TrpA1 channels increased feeding behavior. However, 5-HT1B and 5-HT2A receptor knockdowns significantly decreased mouth hook movements, and 5-HT7 receptor knockdown did not produce significant change in feeding behavior. It was demonstrated previously that blocking 5-HT2A receptors results in a reduction of feeding behavior [9] which concurs with our studies. 5-HT1B receptor knockdown might increase the branching pattern of the 5-HT neurons to locate a target which can provide feedback regulation thereby increasing 5-HT release as volume transmission and suppressing feeding behavior. 5-HT2A and 5-HT7 mutant larvae significantly decreased mouth hook movements. However, Trh mutant larvae did not show a significant reduction in feeding behavior. The reduction in 5-HT7 mutant feeding behavior might be due to the weak musculature structure because the larvae also had a significant reduction in body wall contractions. The reduction of feeding behavior in 5-HT2A mutant larvae confirms the results of 5-HT2A knockdown larvae feeding behavior. 5-HT2B mutant larvae did not show a significant change in feeding behavior compared to w^1118^ larvae. Taken together, our data demonstrate that 5-HT receptors play a role in feeding behavior of* Drosophila* larvae.

### 4.5. Sensorimotor Neural Circuit Modulation by 5-HT

It is already known that 5-HT application to the dissected third-instar larvae increases the evoked sensorimotor circuit activity [34, 38], even though the 5-HT receptor subtypes that mediate modulatory action of 5-HT had yet to be investigated. In the study herein, the results show that 5-HT increased sensorimotor circuit activity at low (100 nM) concentrations but decreased the effect at higher concentrations. This might be due to the desensitization of 5-HT receptors. In attempt to understand which 5-HT receptor subtypes are acting within the neural circuit, the various agonists were applied. For dopamine, it was recently shown that administration of a high concentration itself is toxic to* Drosophila* [66]. The mechanism of this toxicity has yet to be determined. Thus, caution should be considered when exposing the* Drosophila* larvae or adults to high concentration of any biogenic amines.

Since some of the 5-HT agonists responded in a similar fashion to 5-HT, it appears that 5-HT2 and 5-HT7 mediate the modulatory action. Genetic approaches were used to confirm the pharmacological results. 5-HT2A mutant larvae (homozygotes) were less responsive at 100 nM concentration compared to control group; however, 5-HT7 mutant larvae (heterozygote mutants +/−) responded well compared to control group. 5-HT7 mutant larvae (heterozygote mutant +/−) might have enough 5-HT7 receptors to respond well to 5-HT or 5-HT7 receptors might work cooperatively with 5-HT2 receptors to increase the circuit activity. Also, the Trh mutant larvae were very sensitive to exogenous 5-HT application. Since Trh mutant 5-HT neurons have less 5-HT [61], they might compensate for that by increasing sensitivity of the 5-HT receptor expressing neurons to 5-HT. In future studies, it would be good to try some additional agonists and antagonists, such as clozapine, which are known to have a function in other species of insects [15].

We have shown that acute activation of 5-HT neurons by TrpA1 channels decreased locomotion behavior in larvae. However, we do not know the underlying cellular mechanism behind this phenomenon. Therefore, 5-HT neurons were activated by TrpA1 channels while the evoked sensorimotor activity was being recorded. We noted that reduction in locomotion activity is due to the reduction in motor output. Activation of TrpA1 channels in motor neurons, also, resulted in the reduction in locomotive activity but this reduction is due to the spastic contraction of the animals. We have demonstrated that fluoxetine administration reduced locomotion behavior; therefore, we applied fluoxetine to dissected third-instar larvae while evoked sensorimotor activities were being recorded. Fluoxetine at 100 *μ*M significantly reduced sensorimotor activity; however, at a lower concentration, it did not have an effect. A recent study has demonstrated that application of 100 *μ*M of fluoxetine while stimulating channelrhodopsin-2 expressing 5-HT neurons results in blockage of 5-HT reuptake in* Drosophila* larval ventral nerve cord [67]. It has been shown that fluoxetine can block ion channels [68, 69]; therefore, the interpretation of these results is difficult since we have to ensure that the observed effect of fluoxetine is due to the dysregulation of 5-HT level and no other possible nonselective side effects (see [62]). This opens new avenues for the future studies on the mechanism of action of fluoxetine in altering the development in neural circuitry. In order to confirm the effect of 5-HTergic neurons activation by TrpA1 channels, we also used channelrhodopsin-2 to remotely modulate the excitability of 5-HTergic neurons. The results show that 5-HTergic neuron activation, in ChR2-expressing larvae and adults, by blue light exposure significantly decreased locomotion activity in both third-instar larvae and adult flies (Figure 16).

The reason why Canton-S and w^1118^ controls flies showed differences in body wall contractions is not known but has been reported previously [70]. However, it is known that the white gene product is part of tryptophan transporter [71], and tryptophan is the precursor of serotonin. It has been shown that the white gene mutation results in the reduction of 5-HT, dopamine, and histamine content of synaptic vesicles [72]; therefore, the reduction in these biogenic amine levels in w^1118^ larvae might account for the slow locomotion activity compared to CS larvae.

### 4.6. 5-HT Action in Sensory-Motor Circuit Activity: Hypothetical Models

**Figure 18:**
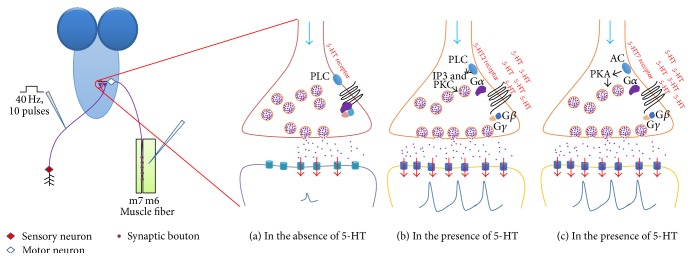
Hypothetical model for the modulatory action of 5-HT in sensory-CNS-motor neural circuitry in* Drosophila* larvae. (a) 5-HT2 receptors are located in the membrane of the presynaptic terminal. In the absence of 5-HT, the strength of synaptic transmission is weak. (b) 5-HT application results in the activation of 5-HT2 receptors which enhances the synaptic transmission due to increment in probability of vesicles release. (c) The activation of 5-HT7 receptors by the 5-HT or 5-HT agonist might activate adenylyl cyclase (AC) starting a cascade of cellular events.

The paradoxical action of exogenous 5-HT application in dissected third-instar larvae and acute activation of 5-HTergic neurons on locomotion activity might be due to the activation of different 5-HT receptors and the degree of 5-HTergic neuron activity. In mammalian system, the action of 5-HT also has shown various responses on spinal motor neuron activity. Low activity of 5-HTergic neurons activates 5-HT2 receptors at the 5-HT-motor neuron synapse which in turn increases the excitability of motor neurons. This results in a higher frequency of motor neuron activity compared to the absence of activity in the 5-HTergic neurons. However, when the 5-HTergic neurons release a large amount of 5-HT, the 5-HT can reach 5-HT1A receptors outside the synapse, the activation of which reduces the activity of sodium channels, and in turn the frequency of action potentials decreases in motor neurons [73]. As previously mentioned, the* Drosophila* genome has five known 5-HT receptor genes. 5-HT1A and 5-HT1B are coupled with G*α* inhibitory (G*α*i) [74]. Activation of 5-HT1A or 5-HT1B receptor subtypes leads to the reduction of cytosolic cAMP level due to the suppression of adenylyl cyclase (AC) enzyme activity. 5-HT7 is coupled with G*α* stimulatory (G*α*s) [75]. When 5-HT7 is activated, it will activate AC that leads to the increasing of cytosolic cAMP. In mammalian systems, 5-HT2 is coupled with the G*α*q protein and has also been shown for the fly* Calliphora vicina* [15] and other insects (e.g., honeybees [76]). When 5-HT binds to 5-HT2 receptors, it will activate phospholipase C (PLC) enzyme [77]. These receptors are expressed in various neurons in multiple regions of the nervous system in* Drosophila* [12]. It has been demonstrated that the sensory neurons of the gill-withdrawal reflex in* Aplysia* can be sensitized by the action of 5-HT, which is released from interneurons. 5-HT binds with various 5-HT receptors which are coupled with either G*α*s or G*α*q. Active G*α*s protein would synthesize cAMP from ATP which in turn activates PKA. In some cells, PKA can lead to blockage of K^+^ channels by phosphorylation. However, active G*α*q can activate PLC which in turn activates PKC, leading to phosphorylation of synaptic vesicle proteins [78]. We present a hypothetical model to explain the molecular mechanism of modulatory action of 5-HT in sensorimotor physiology (Figure 18). The action of 5-HT on neuronal activity is well studied in* Aplysia* model system [79]. Here we hypothesized that 5-HT binds with 5-HT2 receptor on presynaptic terminals activating G*α*q and consequently phospholipase C (PLC), thereby enhancing protein kinase C (PKC). Active PKC can then lead to the established phosphorylation of synaptic vesicle proteins to increase docking of synaptic vesicles. 5-HT might also activate 5-HT7 receptor which activates G*α*s protein, which activates protein kinase A (PKA). Active PKA can also phosphorylate various synaptic vesicle proteins also enhancing the number of docked vesicles and probability of evoked release. Local messenger RNA (mRNA) translation to make proteins, which is important for modulation of synaptic strength, might be another possible mechanism for the modulatory action by 5-HT. It was demonstrated that, during long-term facilitation in an* Aplysia* sensorimotor synapse, mRNAs were being translated at the synaptic site [80].
